# News and Events of the Week

**Published:** 1909-11-06

**Authors:** 


### News and Events of the Week.

Mr. H. T. Butlin, President of the Royal College of Surgeons of England, has been nominated president-elect of the British Medical Association.

The Commission appointed to investigate hook-worm disease in the Southern States of America has received £200,000 from Mr. John D. Rockefeller.

Mr. T. Bates, whose resignation, after thirty years' work as Hon. Surgeon to the Worcester General Infirmary, has been accepted has been asked to accept the post of honorary consulting surgeon to the hospital.

At the Kingston County Bench recently a man was fined £2 and costs for failing, as head of the family, to send notice to the medical officer of health of the district that three of his children were suffering from scarlet fever.

Dr. Lawson, district medical officer to the Marlborough Board of Guardians, was stated at a recent meeting of the Board to have held 29 similar posts in the last 32 years. The Unions in which he has held office are in every part of England.

Dr. R. C. Brown, of Preston, has promised to continue for a further period of two years the scholarship of £150 per annum which he founded in aid of the work of the Cambridge Committee for the Study of Special Diseases on rheumatoid arthritis and allied diseases.

Mr. Jones, surgeon to the Royal Southern Hospital of Liverpool, and lecturer at Liverpool University, has been awarded the Liston Victoria Jubilee Prize (value £100). The prize is given by the Council of the Royal College of Surgeons, Edinburgh, every fourth year to the Fellow or Licentiate of the College who has done the greatest benefit to practical surgery in the four years' interval.

The autumn dinner of the Glasgow University Club, London, will be held at the Gaiety Restaurant, Strand, on Friday, November 5, at 7.30 p.m., when Lord Rosebery, Chancellor of the University, will preside. Members who intend to be present, or to introduce guests, are requested to send notice at once to the honorary secretaries, 4 Bryanston Street, Portman Square, W.

Dr. H. Larder, who recently retired from the post of Medical Superintendent of the Whitechapel Poor-Law Infirmary and Medical Officer of the Workhouse owing to ill-health, has been presented by the Guardians with an illuminated address and a handsome present; and also with a roller-top oak desk and revolving chair by the staff of the Infirmary, Workhouse, and Children's Reception Homes at Bow.

At a meeting of the County Council of Haddington a report was submitted regarding the amalgamation of the offices of county and district medical officers and the officer whom the Secondary Education Committee propose to appoint for the medical inspection of school children. It was recommended that the services of the present county and district medical officers be dispensed with, and that a joint medical officer, holding the Public Health diploma and not to be allowed to engage in private practice, be appointed.

Dr. A. Newsholme has been elected a member of the executive committee of the Imperial Cancer Research Fund, in the place of Dr. P. H. Pye-Smith resigned.

The Bradshaw Lecture was delivered on Tuesday last at the Royal College of Physicians, by Dr. J. A. Lindsay, Professor of Medicine, Queen's College, Belfast. The subject chosen was "Darwinism and Medicine."

At the ordinary quarterly *comitia* of the Royal College of Physicians of London, held last week, the President of the College, Sir R. Douglas Powell, M.D., occupying the chair, the following candidates, having passed the required examinations, were admitted to the membership: W. D. Keyworth, M.B., B.C.Camb., Lieutenant I.M.S., Charing Cross Hospital, and C. J. Singer, M.B.Oxf., L.R.C.P. and M.R.C.S., of St. Mary's.

Among the recent additions to the Library of the Royal College of Physicians are a copy of the very rare first edition of Leopold Auenbrugger's Novum Inventum, Vienna, 1761, the work in which he announced his discovery of the art of percussion of the chest, and an interesting old MS. of the 15th century, containing Latin medical treatises, dating from the end of the 12th and beginning of the 13th century.

At the half-yearly meeting of the General Council of Edinburgh University on October 29, it was agreed to suggest to the University Court the importance of making improvements in the medical curriculum. It was stated that the present curriculum is inadequate, and that many students are taking the course in rather less instead of rather more than five years. To obviate this a suggestion was made that, as formerly, the course should begin in May instead of October.

The Weber-Parkes prize and silver medal of the Royal College of Physicians have been awarded to W. Carnac Wilkinson, M.D.Lond., and a similar medal granted to Arthur Conyers Inman, M.A., M.B., B.Ch.Oxf., the essayist next in order of merit. The prize of the value of 150 guineas was founded in 1895 by Sir Hermann Weber in memory of Dr. E. Alexander Parkes, and is awarded every third year for the best essay upon some subject connected with the ætiology, prevention, pathology, or treatment of tuberculosis, especially in reference to pulmonary consumption in man.

The calendar of the Royal College of Surgeons just published shows that there are 1,457 Fellows on the roll of the College—an increase of 44 over that of last year—of whom 1,415 obtained this distinction by examination, 28 were elected as members of 20 years' standing, and one was elected *ad eundem*. There are 16,957 members and 349 licentiates in midwifery, and 642 holders of the D.P.H. Among the pathological additions to the museum deserving special mention are: The skull of a native of one of the Cook Islands, showing extensive syphilitic disease; portion of the aorta of Menephtah, showing senile calcification; injected lung of a walrus which was drowned, showing communication between the air spaces and blood-vessels. Among the osteological additions are: A collection of 83 crania of a tribe of negroes (Batetala) of the Congo Valley; four crania of ancient inhabitants of Lincolnshire; five crania from the Cook Islands; and a remarkably fine specimen of the skull of the Indian elephant, presented by the London and East India Docks Co.

The Belfast Rural District Council has unanimously adopted Part I. of the Tuberculosis Prevention (Ireland) Act, 1908, which provides for the notification of cases of the disease.

The Executive Committee of the Imperial Cancer Research Fund has requested Lord Crewe to send a circular letter to the Governors of the Colonies and Protectorates, urging the importance of a uniform method of investigation. The committee feels that it is only in this way, for example, that the apparent rarity of cancer in native races can be disproved or explained, and that observations in distant countries can be compared and recorded.

Sir Stephen Mackenzie, a Fellow and former president of the Hunterian Society, consulting physician to the London, the Poplar, and the Royal Ophthalmic Hospitals, left estate of the value of £10,181 gross, and £10,076 net.

The Governor of Mauritius has sent to the Colonial Office the following telegram: "Twenty-one cases of plague reported during week ended October 28. Number of deaths thirteen."

The public introductory lecture of the second year's series given under the auspices of the trustees of the fund endowed by the late Mrs. Honyman Gillespie for research in homœopathy was delivered at the Homœopathic Hospital, Great Ormond Street, last week. The lecturer, Dr. George Burford, took as his subject, "The Medicine of the Future: Coming Events that cast their shadows before," and dealt with the progress of modern medicine. In the absence of the Lady Mayoress, who, with the Lord Mayor, had also promised to be present but was detained at the Mansion House, the chair was taken by Mr. R. Henryson Caird. Mr. Urquhart, one of the trustees of the Honyman Gillesoie Fund, proposed, and Dr. Roberson Day seconded, a vote of thanks to the lecturer, which was carried with acclamation.

A meeting was held last week to inaugurate a guild in connection with the British Medical Benevolent Fund. The object of the new institution is to assist distressed members of the medical profession and their families by personal service and by gifts of clothing and other necessaries. While the fund will deal with money, the guild will give, not money, but money's worth. The following officers were appointed: President, Dowager Lady Broadbent; hon. treasurer, Dr. Mary Thorne; chairman of Council, Lady Tweedy; hon. auditor, Mr. Knox. It was resolved that members should pay an annual subscription of 10s. 6d. and associates 2s. 6d.

